# A Retrospective Trial Exploring Erzhu Yiren Decoction in Gastric Ulcer with Spleen Deficiency and Dampness-Heat

**DOI:** 10.1155/2022/6495181

**Published:** 2022-02-07

**Authors:** Ting Zhang, Zhaowei Shan

**Affiliations:** ^1^Department of Spleen and Stomach Diseases, Nanjing Jiangning Hospital of Traditional Chinese Medicine, Nanjing, China; ^2^Department of Gastroenterology, Jiangsu Province Hospital of Traditional Chinese Medicine, Nanjing, China

## Abstract

**Objective:**

To assess the efficacy of Erzhu Yiren Decoction in treating gastric ulcer of spleen deficiency and dampness-heat and its effect on serum NO, EGF, and PGE2 levels.

**Methods:**

A retrospective study was conducted among 64 patients with gastric ulcers of spleen deficiency and dampness heat admitted to our hospital from September 2019 to May 2020, and they were divided at a ratio of 1 : 1 into an observation group (rabeprazole sodium enteric-coated capsules plus Erzhu Yiren Decoction) and a control group (rabeprazole sodium enteric-coated capsules) based on different treatment methods. The clinical symptom scores, the effective rate of ulcer healing under gastroscopy, the quality of ulcer healing (QOUH), serum nitric oxide (NO), endothelial growth factor (EGF), and prostaglandin E2 (PGE2) levels were compared between the two groups.

**Results:**

The clinical symptom scores of the observation group after treatment were significantly lower than those of the control group (*P* < 0.001). The observation group obtained a remarkably higher efficacy of ulcer healing under gastroscopy than the control group (*P* = 0.039). The observation group outperformed the control group in terms of the number of patients with excellent and good QOUH (*P* = 0.003, 0.014), but no statistical difference in the number of patients with poor QOUH between the two groups was found (*P* > 0.05). Serum NO, EGF, and PGE2 levels of the observation group after treatment were significantly better than those of the control group (*P* < 0.001).

**Conclusion:**

Erzhu Yiren Decoction can relieve the clinical symptoms of patients with gastric ulcers of spleen deficiency and dampness heat; improve the serum NO, EGF, and PGE2 levels; optimize the mucosal maturity; and enhance the overall efficacy, which merits clinical promotion.

## 1. Introduction

Gastric ulcer is a multifactorial disease that is triggered by genetics, gender, smoking, alcohol, and Helicobacter pylori (Hp) infection. Although its pathogenesis has yet been elucidated, it is considered a consequence of the overburdened resistance and downgraded self-repair capability of the gastric mucosa after damages [1–3]. Current Western medicine treatment for gastric ulcers mainly employs drugs that inhibit gastric acid and protect the gastric mucosa and anti-Hp drugs which alleviate the clinical symptoms of patients but also lead to obvious side effects, resulting in a predisposition to more serious diseases [4, 5]. Moreover, clinical studies have shown that Western medicine treatment fails to optimize the quality of ulcer healing (QOUH), with the recurrence rate reaching 80% 1 year after drug withdrawal [6, 7], which is indicative of an unsatisfactory overall efficacy. Nowadays, complementary and alternative medicine (CAM) are commonly used in chronic diseases, including GI disorders [8–12]. Huang et al. provided high-quality efficacy evidence of HXZQ for AD and IBS-D patients and gave an example of postmarketing evaluation for CM products under the pattern dominating different disease research model [8]. It has also been reported that Tangnaikang treatment modulated the abundance of gut bacteria at the class and genus levels, such as Bacilli, Lactobacillus, and Alistipes [10]. Additionally, Khiveh et al. argued that R. ribes syrup can be recommended as a complementary treatment for children with Shigella dysentery [12]. In recent years, traditional Chinese medicine (TCM) has achieved significant results in the treatment of gastrointestinal diseases by virtue of its holistic treatment and syndrome differentiation. Gastric ulcer is categorized into “Piman (distention and fullness)” in TCM [13–15], which is mostly attributed to exogenous factors, emotional abnormality, and irregular diet. Currently, with the acceleration in the pace of life of modern people, the prevalence of gastric ulcers with spleen deficiency and dampness heat has been witnessing a steady increase, the treatment of which therefore has become a hotspot of medical research in China. It has been proposed that Erzhu Yiren Decoction could yield a promising efficacy on gastric ulcers [16], with roasted Astragalus and fried Atractylodes nourishing the qi and invigorating the spleen and stir-fried Atractylodes Rhizome, Magnolia officinalis, stir-fried Scutellaria, and stir-fried Yiren clearing heat and inhibiting dampness, to promote clearness and eliminate the dampness and heat in the stomach and eventually improve the QOUH in patients with gastric ulcer and enhance the protection to the gastric mucosa.

Herein, Erzhu Yiren Decoction was employed to explore its effect on the gastric ulcer with spleen deficiency and dampness heat. The report is as follows.

## 2. Materials and Methods

### 2.1. Research Design

This study is a controlled study and was carried out in our hospital from September 2019 to May 2020, to explore the effectiveness of Erzhu Yiren Decoction in treating gastric ulcers of spleen deficiency and dampness heat and its effect on patients' serum levels of NO, EGF, and PGE2.

### 2.2. Recruitment of Research Subjects

**Figure 1:**
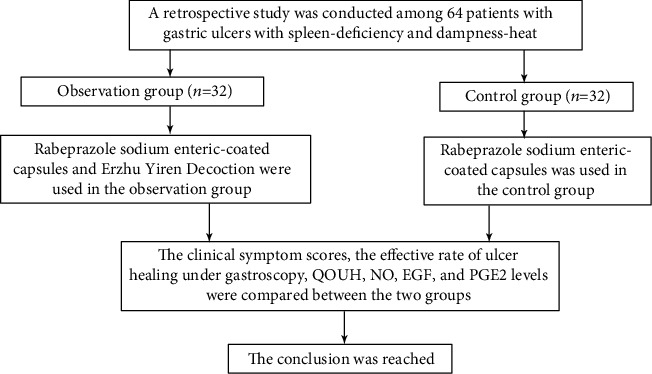
The flowchart of the study.

The data of patients with gastric ulcers of spleen deficiency and dampness heat admitted to our hospital from September 2019 to May 2020 were retrospectively analyzed, and the patients were included as per the eligible criteria: (1) the diagnostic criteria of TCM conformed to the “Guiding Principles for Clinical Research of New Chinese Medicines (2002)” [17] and “Expert Consensus Opinions on the Diagnosis and Treatment of Peptic Ulcer in Traditional Chinese Medicine (2017)” [18]. The diagnostic criteria of Western medicine conformed to the “Standards for Diagnosis and Treatment of Peptic Ulcer Disease (2016)” [19]. Patients with symptoms such as “Piman” (distention and fullness) or pain in the abdomen, dry mouth, or bitter taste in mouth, are diagnosed with active gastric ulcer by gastroscopy within 1 week and met the TCM standard of spleen deficiency and dampness heat; (2) the patients were aged 18-70 years; and (3) no drugs that affect the outcome of ulcers have been used within 1 month before this treatment, such as acid-suppressing antacids, antibiotics, and gastric mucosal protective agents, and no history of drug allergy. The exclusion criteria are as follows: (1) patients with hearing disorders, language disorders, unconsciousness, or mental illness that prevent communication; (2) patients with the withdrawal of consent, death, changes in the treatment plan, or loss to follow-up; (3) pregnant or breast-feeding women; (4) patients with a history of gastrointestinal surgery in the past 3 months; (5) patients with severe liver and kidney dysfunction; (6) patients with blood or immune system diseases or malignant tumors; (7) patients who were allergic to the drugs used in this study; and (8) patients who did not meet the inclusion criteria, with poor medication compliance and factors that affected the efficacy or safety of the drugs. The flowchart of the study is shown in Figure 1.

Herein, a total of 64 patients were included and concurrently randomly assigned to the observation group (*n* = 32) and the control group (*n* = 32). After randomization, the sociodemographic data and clinical performance data of the patients were collected for analysis, and no statistical differences were found in the baseline features between the two groups (*P* > 0.05), as shown in Table 1.

The study was approved by our hospital Ethics Committee (approval no.2018-212), and the patients and their families were informed of the purpose and process of the experimental study and signed the informed consent. This study complied with the principles of the Declaration of Helsinki [20].

### 2.3. Methods

All patients were treated with rabeprazole sodium enteric-coated capsules (specification: 20 mg × 7 s, NMPA Approval Number: H20061220, manufacturer: Jichuan Pharmaceutical Group Co., Ltd.), 20 mg/time, 2 times/day.

The observation group additionally received Erzhu Yiren Decoction (prescription: stir-fried Atractylodes, stir-fried Atractylodes macrocephala Koidz, stir-fried Scutellaria, Roasted Astragalus, Magnolia Officinalis 10 g each, Raw Yiren, Agrimony, stir-fried Squid Bone, Lily 15 g each, and Bletilla striata 6 g). In case of other symptoms, the following symptomatic treatment was given, including stir-fried Ark shells and Bulbus Fritillaria Thunbergii for acid reflux and cardialgia, white peony, Salvia, Aspongopus, Rhizoma Corydalis for severe stomachache, Bergamot, Citrus aurantium for bloating, roasted endothelium Corneum Gigeriae Galli, stir-fried grain malt for poor appetite, Polygonatum, Dendrobium for dry mouth, Herba Eupatorii and Rhizoma Acori Graminei for bad breath, Raphanus seed, Cassia seed for dry stool, Coptis, Aucklandia lappa Decne, and purslane for loose stool and incomplete emptying, Fallopia multiflora Harald, and stir-fried Longgu-Muli for insomnia. This prescription was uniformly prepared by the Chinese pharmacy of our hospital (Jiangsu Wuxi Jiangyin Tianjiang Pharmaceutical Co., Ltd.), one dose per day, dissolved by 200 mL of warm water, with every 100 mL 30 minutes after breakfast and after dinner.

The treatment spanned two months, with two months as a course of treatment.

### 2.4. Outcome Measures

The following are the outcome measures:

1. Baseline features: the number of patients, name, gender, age, average body weight, BMI, course of disease, ulcer diameter, place of residence, monthly income, living habits, and education level are the baseline features
2. Scoring criteria for clinical symptoms: with reference to “Guiding Principles for Clinical Research of New Chinese Medicines” and “Expert Consensus Opinions on Diagnosis and Treatment of Peptic Ulcer in Traditional Chinese Medicine (2017)”, the main symptoms and secondary symptoms before and after treatment were divided into four levels: none, mild, moderate, and severe as per actual clinical symptoms, and were quantified by points. Symptoms and signs were scored (vessels of the tongue were excluded). The main symptoms were recorded as 0, 2, 4, and 6 points from none, mild, moderate, or severe, and secondary symptoms were recorded as 0, 1, 3, and 5 points from mild, moderate, and severe
3. The efficacy of ulcer healing under gastroscope: according to “Consensus on Diagnosis and Treatment of Peptic Ulcer Integrated Traditional Chinese and Western Medicine (2017)”: (i) healed—the ulcer disappeared and the surrounding mucosa was normal; (ii) markedly effective—the ulcer has basically disappeared, and there were still obvious inflammations such as congestion; (iii) effective—the ulcer surface was reduced by more than 50%, but did not disappear; and (iv) ineffective—the ulcer surface was reduced by less than 50%, or there was no change before and after: healing rate = (healed + markedly effective + effective)/total number of cases∗100%
4. QOUH [21]: after treatment, the mucosal changes at the ulcer were observed under the endoscope, to evaluate the maturity of the regenerated mucosa. Two mucosal tissues were taken from the center of the scar for hematoxylin-eosin (HE) staining, and routine pathological observation was performed to evaluate the histological maturity of the regenerated mucosa
5. Serum levels of nitric oxide (NO), endothelial growth factor (EGF), and prostaglandin E2 (PGE2): early morning fasting venous blood was collected from all patients before treatment (T1), 1 month after treatment (T2), and 2 months after treatment (T3), centrifuged at 3000 r/min for 10 min to obtain the serum. Nitrate enzyme reduction method (MitoSciences, original supporting reagents, NMPA Approval Number 3402843) was used to determine NO, and enzyme-linked immunosorbent assay (Beijing Kewei Clinical Diagnostic Reagent Co., Ltd., enzyme-linked immunosorbent kit, NMPA Approval Number S20060028) was used to determine the content of EGF and PGE2

### 2.5. Statistical Analyses

The data processing software selected in this research was SPSS20.0, and GraphPad Prism 7 (GraphPad Software, San Diego, USA) was used to plot the graphics. The counting data were expressed by *n* (%) and processed using the chi-square test, and the measurement data were expressed by $\bar{x}\pm s$ and analyzed by a *t*-test. *P* < 0.05 indicates that the difference is statistically significant.

## 3. Results

### 3.1. Comparison of Baseline Features

The two groups had no significant difference in baseline features (*P* > 0.05). See Table 1.

### 3.2. Comparison of Clinical Symptom Scores

Erzhu Yiren Decoction plus rabeprazole sodium enteric-coated capsules obtained remarkably lower clinical symptom scores after treatment versus rabeprazole sodium enteric-coated capsules alone (*P* < 0.001). See Table 2.

### 3.3. Comparison of the Efficacy of Ulcer Healing under Gastroscopy

The observation group obtained a significantly higher efficacy of ulcer healing under gastroscopy than the control group (*P* < 0.05). See Table 3.

### 3.4. Comparison of QOUH

**Figure 2:**
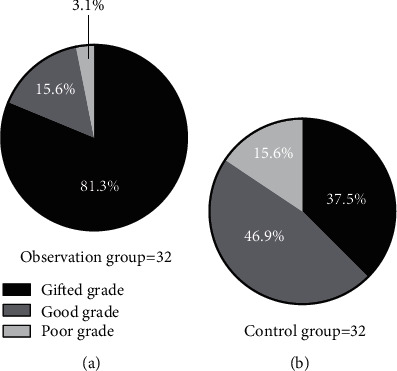
Comparison of the maturity of the mucosal tissue structure around the gastric ulcer after treatment [*n* (%)]. Note: (a) is the observation group, and (b) is the control group; the black area in the figure is excellent QOUH, the dark gray area is good QOUH, and the light gray area is poor QOUH. The observation group was statistically different from the control group in terms of the number of patients with excellent and good QOUH (26 vs. 12, 5 vs. 15, *P* < 0.05); no statistical difference in the number of patients with poor QOUH between the two groups was found (1 vs. 5, *P* > 0.05).

The observation group outperformed the control group in terms of the number of patients with excellent and good QOUH (*P* < 0.05), and but no statistical difference in the number of patients with poor QOUH between the two groups was found (*P* > 0.05). See Figure 2 and Table 4.

### 3.5. Comparison of Serum NO, EGF, and PGE2 Levels

**Figure 3:**
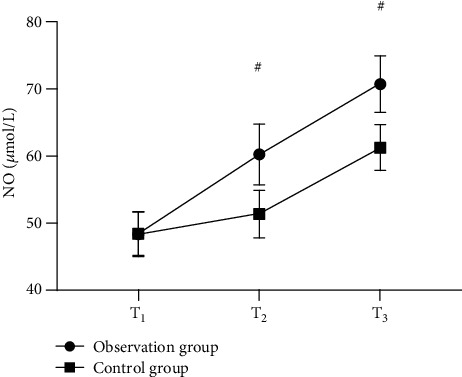
Comparison of serum NO levels in patients ($\bar{x}\pm s$, *μ*mol/L). Note: the horizontal axis of the figure from left to right is before treatment (T1), 1 month after treatment (T2), and 2 months after treatment (T3). The dotted line in the figure is group A, and the square line is group B; # indicates *P* < 0.001. The serum NO level of T1 in the observation group and the control group was not statistically different (48.32 ± 3.35 vs. 48.40 ± 3.21, *P* > 0.05); serum NO levels of T2 and T3 in the observation group were significantly higher than those in the control group (60.21 ± 4.52 vs. 51.32 ± 3.54, 70.68 ± 4.21 vs. 61.23 ± 3.41, *P* < 0.001).

**Figure 4:**
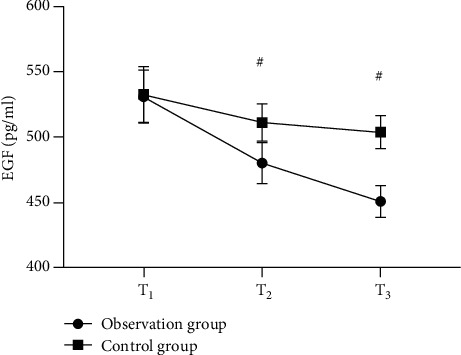
Comparison of serum EGF levels in patients ($\bar{x}\pm s$, pg/ml). Note: the horizontal axis in the figure from left to right is before treatment (T1), 1 month after treatment (T2), and 2 months after treatment (T3). The dotted line in the figure is group A, and the square line is group B; # indicates *P* < 0.001. The serum EGF levels of T1 in the observation group and the control group were not statistically different (530.98 ± 20.24 vs. 532.68 ± 21.25, *P* > 0.05); serum EGF levels of T2 and T3 in the observation group were significantly lower than those in the control group (480.22 ± 15.68 vs. 511.26 ± 14.23, 450.85 ± 12.10 vs. 503.98 ± 12.68, *P* < 0.001).

**Figure 5:**
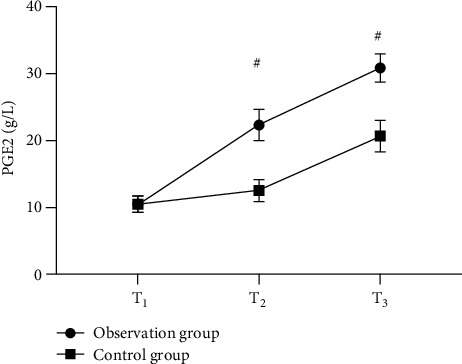
Comparison of serum PGE2 levels in patients ($\bar{x}\pm s$, g/L). Note: the horizontal axis in the figure from left to right is before treatment (T1), 1 month after treatment (T2), and 2 months after treatment (T3). The dotted line in the figure is group A, and the square line is group B; # indicates *P* < 0.001. The serum PGE2 levels of T1 in the observation group and the control group were not statistically different (10.54 ± 1.22 vs. 10.56 ± 1.23, *P* > 0.05); serum PGE2 levels of T2 and T3 in the observation group were significantly higher than those in the control group (22.32 ± 2.32 vs. 12.58 ± 1.65, 30.82 ± 2.10 vs. 20.68 ± 2.35, *P* < 0.001).

Strong evidence of better serum NO, EGF, and PGE2 levels of the observation group after treatment than those of the control group was found (*P* < 0.001). See Figures 3–5.

## 4. Discussion

Gastric ulcer is a common clinical disease with elusive pathogenesis, which involves influencing factors such as genetics, environment, and Hp. Common symptoms include dry mouth, nausea, abdominal pain, gastric bleeding, and gastric perforation and even cause malignant lesions which are associated with severe cases, which compromise the life and health of the patients. Currently, Western medicine is frequently used for the treatment of gastric ulcers in clinical practice; however, previous research has pointed out that Western medicine treatment fails to achieve long-term curative effects, with a high recurrence rate [22] and a poor quality of ulcer healing. Tarnawski believes that repairing and reconstructing submucosal tissue is the key to the treatment of gastric ulcers. Clinically, a remnant of pathological changes still exists in some of the healed ulcers observed by endoscopy, which gives weight to the determination of functional maturity of the ulcers in addition to the observation of superficial regenerated mucosa under direct endoscopy, to better evaluate the recurrence of the disease [23]. A prior study has applied Hewei Xiaoyang Decoction in the treatment of gastric ulcer and found a significantly improved maturity level of the mucosal tissue structure around the gastric ulcer [24], indicating that the holistic treatment and the concept of syndrome differentiation of TCM yield a positive efficiency.

Gastric ulcer is categorized into “Piman (distention and fullness)” in TCM, with the medical syndrome classification mainly including deficiency of the spleen and stomach, stagnation of Qi in the liver and stomach, dampness heat in the spleen and stomach, stagnation of heat in the liver and stomach, stagnation of qi and blood stasis, deficiency and cold of the spleen and stomach, and yin deficiency of the stomach. TCM believed that the deficiency and cold of the spleen and stomach are the main pathogeneses of gastric ulcer, with the treatment centering on the reinforcement of the spleen and replenishment of Qi, which has achieved promising clinical efficacy. Nevertheless, with the acceleration of the pace of life of modern people, unhealthy living habits and dietary patterns such as preference for spicy, fat, sweet, thick, and greasy food, or addiction to smoking and alcohol, have resulted in alterations in clinical syndrome differentiation, just as in a TCM classic [25]: “most patients with stomachache prefer spicy and fatty foods or alcohol.” In recent years, data show that the number of patients with gastric ulcers with spleen deficiency and dampness heat is presenting a trend of gradual increase, which has become a research hotspot in China. The Erzhu Yiren Decoction employed in this study was proposed by scholar Shan Zhaowei, who points out that damp-heat syndrome is more common in the onset of ulcers with basic pathogenesis due to spleen deficiency. Specifically, deficiency of the spleen fails to drain out the dampness and results in the heat for the long run, spleen and stomach dampness and heat, disharmony of the middle jiao, and damage to the stomach collaterals, which ultimately trigger ulcers. The roasted Astragalus and stir-fried Atractylodes macrocephala Koidz in Erzhu Yiren Decoction invigorate the spleen and replenish Qi, and the stir-fried Squid Bone and Bletilla striata yield an excellent acid-inhibiting effect. Moreover, the combination with Lily with a moisturizing effect succeeds in replenishing Qi and invigorating the spleen, clearing away heat and dampness, and eventually yields a gastric-protective efficacy [26, 27]. Therefore, the effective rate of gastroscopic ulcer healing in the observation group was significantly higher than that of the control group (*P* < 0.05), and the clinical symptom score after treatment was significantly lower than that of the control group (*P* < 0.001), indicating significantly improved gastric ulcer after the introduction of Erzhu Yiren Decoction.

From the perspective of modern pharmacology, the baicalein in Scutellaria effectively regulates interleukin-1*β* and accelerates the production of PGE2. Research by Ji et al. showed that Dahuang-Huanglian-Xiexin Decoction based on rhubarb and Scutellaria could increase the level of PGE2 in the gastric tissue of rats with gastric ulcers [28], enhance the gastric mucosal protective mechanism, reduce gastric acid secretion, and inhibit the release of cytotoxic substances and lysosomal enzymes, resulting in a better gastric mucosal repair [29, 30]. Roasted Astragalus regulates the secretion of Th-type cytokines such as tumor necrosis factor and interleukin-1 and mitigates their damage to endothelial cells, facilitating the synthesis and release of NO from vascular endothelial cells. In this study, the combination of the aforementioned TCM herbs successfully drove down the patients' serum NO, EGF, and PGE2 levels; improved the QOUH in the observation group; and contributed to the reduction of disease recurrence.

In conclusion, Erzhu Yiren Decoction can relieve the clinical symptoms of patients with gastric ulcers with spleen deficiency and dampness heat; improve the serum NO, EGF, and PGE2 levels; optimize the mucosal maturity; and enhance the overall efficacy, which merits clinical promotion.

## Figures and Tables

**Table 1:** Comparison of general information.

Groups	Observation group (*n* = 32)	Control group (*n* = 32)	*χ* ^2^/*t*	*P*
Gender			0.159	0.611
Male	20	18		
Female	12	14		
Age (year)				
Range	26-64	27-65		
Average age	45.26 ± 2.65	45.25 ± 2.14	0.017	0.987
Average weight (kg)	55.10 ± 2.11	55.15 ± 2.13	0.094	0.925
BMI (kg/m^2^)	21.23 ± 2.14	21.41 ± 2.10	0.340	0.735
Course of disease (year)				
Range	1-9	2-8		
Average course of disease	5.21 ± 1.22	5.24 ± 1.20	0.099	0.921
Ulcer diameter (cm)				
Range	0.6-1.5	0.7-1.4		
Average diameter	0.89 ± 0.13	0.92 ± 0.12	0.959	0.341
Place of residence			0.251	0.616
Urban	16	14		
Rural	16	18		
Monthly income (yuan)			0.063	0.802
≥4000	15	16		
<4000	17	16		
Living habits				
Smoking	12	13	0.066	0.798
Drinking	10	12	0.277	0.599
Education level			0.063	0.802
High school and below	16	15		
University and above	16	17		

**Table 2:** Comparison of clinical symptom scores of patients ($\bar{x}\pm s$, points).

Groups	Observation group (*n* = 60)	Control group (*n* = 60)	*χ* ^2^/*t*	*P*
Abdominal fullness or pain				
Before treatment	4.21 ± 1.23	4.23 ± 1.20	0.066	0.948
After treatment	1.10 ± 0.54	2.11 ± 0.68	6.580	<0.001
Dry mouth or bitter taste				
Before treatment	4.56 ± 0.89	4.61 ± 0.78	0.239	0.812
After treatment	1.34 ± 0.21	2.23 ± 0.57	8.288	<0.001
Dry mouth without the desire to drink				
Before treatment	3.91 ± 0.41	3.95 ± 0.35	0.420	0.676
After treatment	0.74 ± 0.35	1.56 ± 0.24	10.930	<0.001
Limb fatigue				
Before treatment	4.00 ± 0.65	4.05 ± 0.57	0.327	0.745
After treatment	1.00 ± 0.21	1.68 ± 0.23	12.351	<0.001
Poor appetite				
Before treatment	3.45 ± 0.35	3.46 ± 0.36	0.113	0.911
After treatment	0.67 ± 0.12	1.23 ± 0.20	13.582	<0.001
Nausea or vomiting				
Before treatment	3.78 ± 0.45	3.70 ± 0.50	0.673	0.504
After treatment	0.89 ± 0.12	1.45 ± 0.23	12.211	<0.001
Oliguria				
Before treatment	3.56 ± 0.35	3.60 ± 0.45	0.397	0.693
After treatment	0.88 ± 0.15	1.35 ± 0.23	9.682	<0.001
Loose stool				
Before treatment	3.24 ± 0.43	3.30 ± 0.42	0.565	0.574
After treatment	0.74 ± 0.14	1.33 ± 0.32	9.555	<0.001

**Table 3:** Comparison of the efficacy of gastroscopy in patients [*n* (%)].

Groups	Healed	Markedly effective	Effective	Ineffective	Total effective rate
Observation group	10 (31.3)	12 (37.5)	8 (25.0)	2 (6.3)	30 (93.8)
Control group	3 (9.4)	10 (31.3)	12 (37.5)	8 (25.0)	24 (75.0)
*χ* ^2^	4.730	0.277	1.164	4.267	4.267
*P*	0.030	0.599	0.281	0.039	0.039

**Table 4:** Maturity of tissue structure of regenerated mucosa in patients with Hp eradication and non-eradication after treatment [*n* (%)].

Groups	Observation group (*n* = 32)	Control group (*n* = 32)	*χ* ^2^/*t*	*P*
Hp eradication (case)	28	28		
Excellent	20 (71.4)	8 (28.6)	8.654	0.003
Good	7 (25.0)	16 (57.1)	5.976	0.014
Poor	1 (3.6)	4 (14.3)	1.977	0.160
Hp noneradication (case)	4	4		
Excellent	3 (75.0)	2 (50.0)	0.533	0.465
Good	1 (25.0)	2 (50.0)	0.533	0.465
Poor	0 (0.0)	0 (0.0)	0.000	1.000

## Data Availability

The datasets used and analyzed during the current study are available from the corresponding author on reasonable request.
